# A Review on Different Kinds of Artificial Intelligence Solutions in TCM Syndrome Differentiation Application

**DOI:** 10.1155/2021/6654545

**Published:** 2021-03-09

**Authors:** Yujuan Song, Bin Zhao, Jun Jia, Xuebing Wang, Sibai Xu, Zhenjing Li, Xu Fang

**Affiliations:** ^1^TCM Department, Shenzhen Longhua District Central Hospital, Guanlan Avenue No. 187, Shenzhen, Guangdong, China; ^2^Rehabilitation Department, The Second Affiliated Hospital of Heilongjiang University of Traditional Chinese Medicine, Harbin, Heilongjiang, China; ^3^Rehabilitation Department, Shenzhen Longhua District Central Hospital, Guanlan Avenue No. 187, Shenzhen, Guangdong, China; ^4^Rehabilitation Department, Hannover Medical School, Carl-Neuberg-Str. 1, Hannover 30625, Germany; ^5^Human Resources Department, Shenzhen Longhua District Central Hospital, Guanlan Avenue No. 187, Shenzhen, Guangdong, China

## Abstract

In 1979, the first computer program for TCM diagnosis was launched, although this time was about 30 years after artificial intelligence (AI) came into being and began to be widely used. However, an endless stream of artificial intelligence methods was applied in the field of Chinese medicine research, expert system, artificial neural network, data mining, and multivariate analysis; not limited to what was mentioned, this study tried to make a review on application of AI to TCM syndrome differentiation, while summarizing the artificial intelligence application of TCM syndrome differentiation in the current context. It also provides a theoretical background for the upcoming fully automated research on TCM syndrome differentiation and diagnosis robot.

## 1. Introduction

TCM diagnostics is one of the basic theories of TCM (Traditional Chinese Medicine), aimed at examining the clinical manifestations of symptoms in patients, so as to make a definite syndrome diagnosis and analyze the progression of diseases [1]. TCM diagnosis has two important characteristics. One is the concept of holism; i.e., the human body is an organic whole, where all the organs and tissues are inextricably linked with one another. Local changes within the human body reflect its constitutional conditions, while internal changes are manifested outwardly. During the process of clinical diagnosis and treatment, a traditional Chinese physician infers a pathological change in the human body based on the patient's experience and his own observation. The other is syndrome differentiation and treatment, which is the essence of TCM and the basis and prerequisite of all TCM-related clinical activities. Syndrome differentiation is the core of TCM diagnosis and is characterized by differentiation of disease and differentiation of syndrome, of which differentiation of disease comes prior to differentiation of syndrome [2, 3]. Only correct differentiation of the various syndromes is able to ensure reasonable treatment of diseases [4].

The physician's own knowledge of the TCM theory and accumulated clinical experience is relied upon to make a TCM diagnosis using the four diagnostic methods and differentiation methods, according to the patients' clinical symptoms [1]. Subjective factors play a decisive role in TCM diagnosis. This unique diagnostic mode promotes the open and divergent development of TCM; however, it has become a bottleneck to its development. The accuracy of diagnostic results is too dependent on the traditional Chinese physicians' ability and experience, owing to the difference in personal competence among traditional Chinese physicians; therefore, the therapeutic effect of TCM is inevitably uncertain [4].

In the 1950s, artificial intelligence (AI) came into being and began to be widely used in various disciplines [5]. In 1979, the first computer program for TCM diagnosis was launched, initiating the development of an intelligent TCM diagnostic system. In the 1980s, rule-based decision inference was widely used in expert systems, significantly characterizing the intellectualization of TCM diagnosis during this period. On the one hand, a knowledge tree was built to make inferences by judging the credibility of nodes, while a backtracking mechanism was used to achieve optimization (or knowledge was absorbed inversely according to the production rule); on the other hand, the matrix and array were taken as forms of knowledge expression, while extremum searching was used to build a heuristic inference engine. Moreover, multiple inference techniques started being combined in strict accordance with TCM holistic thinking [6]. In 1989, Prof. Qin, a bioengineer at the Capital Medical University, published *An Introduction to the Computer Simulation and Expert System of TCM*, where representative TCM expert systems are classified and summarized from the perspective of thinking models and techniques. A clue as to the replacement of expert systems with intelligent syndrome differentiation was discovered at that time. However, the studies conducted during this period of time were severely restricted by the concept of these early expert systems, e.g., it is difficult to acquire knowledge, and studies must depend on complete expert knowledge; the combination of diseases syndromes and symptoms is not yet universal, and the self-learning ability of the systems and complexity and specificity of diseases was ignored. Excessive energy was used to study and arrange expert knowledge and inference rules, while there was not sufficient attention to the diversity and uncertainty of applications in clinical practice. In the 1990s, mathematical inference, analysis, and statistical methods were put into use, promoting the development of diagnostic inferences in TCM; however, the linear inference mode could not cope with highly nonlinear characteristics of TCM syndromes [7]. In recent years, a series of methods have been put forward for dealing with nonlinear technical problems, such as soft computing and data mining. For example, Gan applied the fuzzy mathematics theory to TCM diagnosis [8]; Zhang Qin et al. made a combined use of the cluster analysis and fuzzy theory to extract a number of syndrome principles concerning hepatopathy in TCM clinical practice, presenting a method for syndrome identification [9]; Qin and Mao applied an improved neural network model based on a rough set to the typical diagnosis of rheumatoid diseases, greatly increasing the accuracy of diagnosis [10]; advanced smart technology was applied to TCM syndrome differentiation, breaking through the expert system, improving the flexibility and practicability of the diagnostic system, lifting the attachment of the rules and differentiation system to complete knowledge and linear programming, finally enhancing the independence of the system and its ability to solve nonlinear problems. The diagnosis model built on the basis of smart technology greatly reduced the influence of various subjective factors on the development of TCM syndrome differentiation, dramatically promoting the intellectualization of TCM syndrome differentiation [5, 6].

Intelligent diagnosis of TCM has grown out of a computer-based combination of AI technology and the TCM diagnosis theory. The emergence and development of each smart technology features its own unique knowledge background and research direction. As such, each must have advantages and disadvantages in practical applications.

## 2. Expert System (ES)

**Figure 1:**
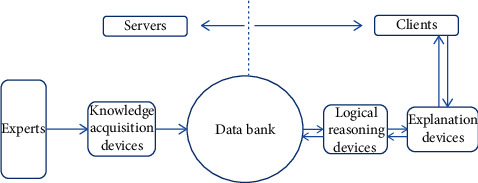
Expert system schematic.

The ES was the embryonic form of AI and laid a foundation for the generation and development of AI, promoting the transformation of intellectualization from a textual theory into a practical application, bringing about a breakthrough in replacing mechanical logical inference with the use of expert knowledge to solve complex problems. Its advantages are as follows: it could handle complex system problems difficult to express and statistically analyze in a mathematical model; and the expression of knowledge was readable and easy to understand. Its disadvantages are as follows: it had difficulty in acquiring tacit knowledge and could not give a clear instruction description of uncertain problems; the ES did not have the ability to learn independently, while its development depended on a complete knowledge base; the knowledge was so subjective that an inaccurate conclusion might be drawn for some problems, and the conflict between different expert knowledge could not be solved; because the knowledge came from a limited scope and field, it could not play a part once a problem was beyond said scope or field; moreover, as there was more and more knowledge in the ES, the size of the knowledge base would be enlarged infinitely, thus reducing the efficiency of inferences. The serial treatment method had great difficulty in solving parallel problems. The ES of TCM syndrome differentiation could help to make a logical inference about TCM syndrome differentiation based on TCM experts' rich clinical experience and knowledge under a fully transparent inference rule [11, 12]. However, when used in a complex clinical medical diagnosis environment, the ES called for the solving of difficult technical problems, such as bottle-necked knowledge acquisition, the lack of memory and self-learning ability, and the nonrobustness of the system, limited to rules as it is. All these problems made it difficult for the ES to be suitable for the inference and calculation of heterogeneous, high-dimensional, and fuzzy clinical TCM data [9]; see Figure 1.

## 3. Artificial Neural Network (ANN)

**Figure 2:**
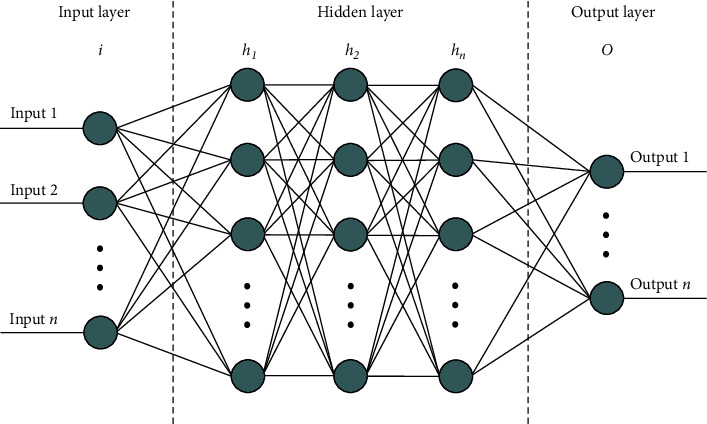
Artificial neural network architecture [17].

ANN is another important research field of AI. Different from the rule-based ES, ANN is a physiological bionic operation model that works by simulating the activity of the cerebral neural network. It is a nonlinear and self-adaptive dynamic system formed from the adjusted connection between nodes (neurons) [13, 14]. The basic unit of its structure is the neuron (i.e., the information node). Each neuron is composed of three parts: cell body, dendrites, and axons. The connection points between independent neurons are synapses [15]. The neurons transmit and process the information resources in the network through weighted interconnection [16]. A neuron can convert the information from other neurons connected to it into a self-input activation function to calculate the corresponding output information, and then send the information to other neurons. The neural network and its simplified structure are shown in Figure 2 [17]. There are many types of ANN models, such as Rosenblatt's perceptron model and BP network machine algorithm, Widrow's ADALINE model and MADALINE model, Grossberg's ART model, Kohonen's SOM model, Hinton's Boltzmann machine, Hopfield's Hopfield network, and Rumelhart's PDP model; see Figure 2.

The ANN has multiple advantages in simulating the activity of the human brain, including parallelism, nonlinearity, and nonconvexity. It is fairly able to handle practical application problems characterized by a complex information environment and fuzzy or unclear knowledge and rules. With its self-organizing ability, adaptive ability, and strong self-learning ability, it can adjust and change its own connection weight under constantly changing external information. The state of the neurons and the connection between them determine the holistic system activity. However, the ANN also has many shortcomings: Its “black box” mode makes the neural network unable to explain the knowledge and the entire inference process; the learning of the neural network requires a lot of samples, thus prolonging the training duration. Moreover, local minimum and overfitting are hard to avoid [18]. According to the above description and analysis of the characteristics of the neural network, it is not difficult to find that many of its characteristics are very similar to the highly fuzzy and nonlinear knowledge structure of TCM, as well as the logical inference of TCM syndrome differentiation. By independently learning clinical information resources, it is able to discover the complex connection between the symptom and syndrome without relying on the formalized description and clear rules and mechanisms of the mathematical model. This fully displays the flexibility and adaptive ability of the network. Therefore, the ANN is a good tool and method in the process of intelligent TCM syndrome differentiation [16]. However, the susceptibility of the ANN to local minimum and overfitting affects the accuracy and reliability of clinical diagnosis, making it impossible to realize rule integration and clinical practice guidance. To adapt to the characteristics and requirements of TCM syndrome differentiation, it can be combined with other intelligent technologies and methods to reduce the impact of its own deficiencies on syndrome differentiation.

## 4. Data Mining (DM)

**Figure 3:**
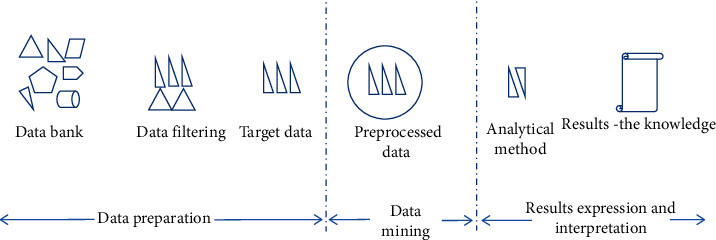
Data mining process.

DM, also called Knowledge Discovery in Database (KDD), is a smart technology that is used to extract relevant laws and rules from massive data by analyzing various data [19]. The basic process of DM is shown in Figure 3.

DM technology, which is completely free from the pyramidal cascading research model, focuses on researching the knowledge contained in real data; as such, the laws revealed are objective and reliable. From the perspective of information science and technology [20], the research of intelligent TCM syndrome differentiation is a process from data to discovery, involving data acquisition, data management, data analysis, and simulation, as shown in Figure 3 [21, 22]. DM technology is also a feasible method suitable for intelligent TCM syndrome differentiation. Through DM, syndromes can be differentiated objectively and quantitatively. This indicates that it is a good mode of research on the laws of TCM syndrome differentiation [23]; see Figure 3.

From the perspective of laws and knowledge acquisition, almost all DM techniques are related to machine learning [11, 24]. Unlike the traditional methods such as cluster analysis and regression analysis, which are performed in order to obtain quantitative data features from data, the decision tree, association analysis, and Bayesian learning help to acquire object-oriented knowledge [25, 26].

**Figure 4:**
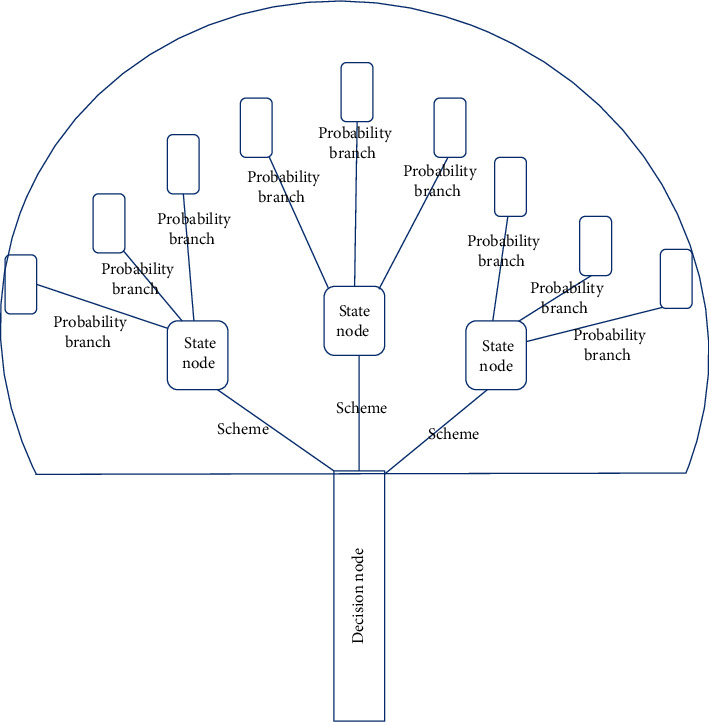
Decision tree element composition diagram.

The decision tree is a tree structure which solves the problem of syndrome differentiation and classification in TCM. As shown by the essential elements of the decision tree in Figure 4, with a simple structure and high classification efficiency, it can process and analyze a large amount of data concerning nonlinear relationships to reveal relevant attributes and rules, which are mostly used for research on syndrome classification [27, 28]. ID3, C4.5, SLIQ, and SPRINT, etc. are all typical algorithms in the decision tree [19]; see Figure 4.

**Figure 5:**
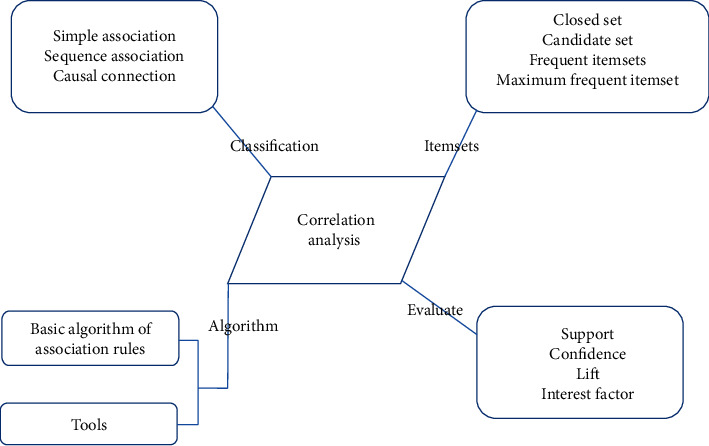
Rules schematic correlation analysis.

Association analysis is a process of obtaining effective knowledge with specific minimum support and minimum confidence. The association rules reflect the dependency and association among different categories of things, as shown by the diagram of association analysis rules in Figure 5. These two methods have also been widely used in practice; see Figure 5.

Wang and Ma studied the diagnosis of hepatopathy after its periodic occurrence in TCM clinical practice, optimizing and improving the decision tree algorithm, discovering a law by which the pathogenetic condition could be diagnosed periodically, thus differentiating compensated cirrhosis from decompensated cirrhosis clearly and quantitatively [28]. Yao et al. made some preliminary studies on the compound compatibility of Chinese herbal drugs based on pharmacology and toxicology using the association analysis technology and verified the results in hundreds of TCM cases treated [29]. Li et al. discovered the application of frequent item sets in pharmacology and drug efficacy through DM, extracting the association rules regarding drug interactions based on the data about many years of clinical cases with infectious hepatopathy [30]. The advantages of the decision tree and association analysis are obvious [31, 32]. Their strong generalization ability makes it easier to extract rules. Moreover, the rules extracted are usually easy to explain and understand; other important attributes can be extracted if the rules extracted are further learned; furthermore, the computational complexity can absolutely be estimated and predicted. However, the decision tree and association analysis are instable, so the difference in results obtained from training samples and test samples should not be underestimated; if some data items are missing, the classification effect will be directly affected; the database is scanned and sorted too frequently in the process of rule extraction, producing a large number of intermediate results, which affects the processing speed of larger training samples.

DM is suitable for solving and revealing the hidden connections within data and the acquisition of effective knowledge. However, in the process of building an intelligent system of TCM syndrome differentiation, the extensive and simple application of DM technology is obviously unable to cope with high fuzziness, high dimensionality, nonlinearity, and uncertainty when they are interwoven together. In TCM clinical practice, various signs, which are related to symptoms and syndromes, are independent and interdependent on one another. This diversity and complexity determine a specific knowledge structure system, making it difficult to set a model expression expectation in line with its features effectively and accurately through DM [33, 34].

## 5. Multivariate Analysis

**Figure 6:**
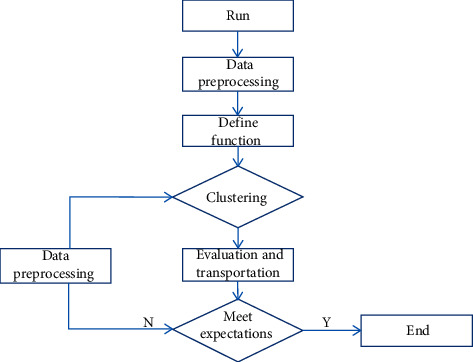
Cluster analysis process.

Multivariate analysis, also called multifactor analysis, is a common statistical method. It is mainly used to study the relationship among several interdependent factors and the relationship among the samples with such characteristics [35]. In TCM syndrome differentiation, such dependency-based multivariate analyses can be roughly divided into two types: the first type is characterized by dependency among various factors. The relevant processing methods include principal component analysis (PCA), factor analysis, cluster analysis, etc. Its goal is to obtain the corresponding symptom dataset and explain it. The main process of cluster analysis is shown in Figure 6.

**Figure 7:**
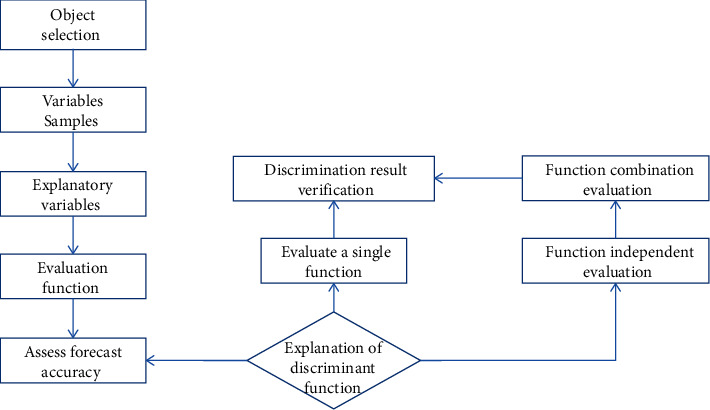
Discriminatory analysis process.

However, it dilutes the implicitness and ambiguity of the internal relation between “symptom” and “syndrome” [36]. The high-dimensional complexity, i.e., they are not only intertwined with each other, but also separated from each other, makes it difficult for this type of method to reveal the clear structure of the TCM clinical data. For example, most patients may have multiple secondary symptoms, and a single symptom may have specific clinical manifestations in different syndromes. In other words, an individual patient and his/her symptoms are very likely to be clustered into multiple categories. However, according to the one-to-one classification of cluster analysis, it is obviously preposterous that a certain symptom or individual is put under a single category. Thus, it has extreme limitations and ambiguity in use, and so, it can only be used as a supplementary means; the other is the dependency between antecedents and consequences; i.e., syndromes (consequences) are dependent on symptoms (antecedents). Multiple linear regression analysis, discriminatory analysis, and logistic regression are the main methods; see Figure 7.

The process of discriminatory analysis is shown in Figure 7. By building a functional relationship and diagnostic inference model that simulates the process of TCM diagnosis, this type of method can reveal the internal hidden relationship between them according to their mathematical relationship. However, there is not just a simple one-to-one relationship between TCM syndrome and symptom, so it is indeed difficult for a single relational function to accurately describe such multidimensional and multilayered relationships. Moreover, it is hard to provide a simple and effective method for rule differentiation, so it has no guiding significance for TCM clinical diagnosis [36]. By combining the TCM theory with clinical practice, we can easily see that the multivariate statistical analysis' ability to express the relationship between complex, multidimensional objects is not suitable for intelligent TCM syndrome differentiation [37].

In addition, AI also contains many other intelligent technologies, such as rough sets, support vector machines, grey theory, and agent, all of which have been widely used in practice [7]. During the intellectualization of the TCM syndrome differentiation system, the fuzzy theory can be used to appropriately deal with the high ambiguity of knowledge structure; the emerging intelligent methods and their improved technologies provide a broader platform for the optimization of diagnostic procedures; the combination of multiple methods and strategies can make up for the shortcomings brought about by the independent application of a certain technology, thus strengthening the system's ability to solve problems [37].

Research on intelligent TCM diagnosis is an inexorable trend in the information age. However, during a hard work of literatures reading, we found that there was a separation of TCM AI relevant research trends in and outside China; as we listed in the reference parts, most TCM AI relevant studies were in China, and published in Chinese; this gap made a big difficult language problem to push AI TCM relevant study forward. It seems only one way by reporting more with international general scientific research language. Meanwhile, the modernization of TCM is of far-reaching significance, combining emerging intelligent techniques with traditional TCM diagnosis to develop AI-based TCM syndrome differentiation technology with the aim of eventually building an intelligent diagnostic system. This is also of great practical significance in improving traditional Chinese physicians' diagnostic capability while further enhancing their training. The application of smart technology in TCM plays a role in promoting the research of the laws of TCM syndrome differentiation, thereby changing the completely arbitrary diagnostic approach, enriching the content and method of TCM studies.

## Data Availability

The data are stored in Guangzhou Medical University Data Center, which is the responsibility of the first author and corresponding author, and will be made public three years later. The data will be stored for 10 years.
